# Evaluating Bone Grafting Success in Maxillofacial Surgery Among Patients With Oral Mucosal Diseases: A Prospective Cohort Study

**DOI:** 10.7759/cureus.92849

**Published:** 2025-09-21

**Authors:** Mariam Fatima, Kashaf Ali, Tayyaba Rafiq, Anil Kumar, Tariq Aziz

**Affiliations:** 1 Oral Medicine, Shifa College of Dentistry, Shifa Tameer e Millat University, Islamabad, PAK; 2 Pathology, Indus Medical College and Hospital, Tando Muhammad Khan, PAK; 3 Oral and Maxillofacial Surgery, Lahore Medical and Dental College, Lahore, PAK; 4 Oral and Maxillofacial Surgery, Muhammad Dental College, Mirpur Khas, PAK; 5 Oral Biology, Muhammad Dental College, Mirpur Khas, PAK

**Keywords:** dental implants, immunosuppressive agents, lichen planus, mouth mucosa, oral, surgery

## Abstract

Background: In maxillofacial surgery, bone grafting plays a critical role in alveolar defect reconstruction and dental bone grafts. Oral mucosal diseases (OMDs), including oral lichen planus and mucous membrane pemphigoid, may impair the local epithelial integrity and immune reactions. The objective of this research was to compare six-month graft integration between immune-mediated OMDs and those without and evaluate wound healing complications, surgical site infections, and factors of graft failure (immunosuppressive therapy, glycemic control, and smoking).

Materials and methods: A total of 86 patients who underwent autogenous or alloplastic bone grafting at a tertiary care hospital were studied between June 2023 and December 2023 as part of a prospective cohort study, with outcomes assessed at six months. The participants were divided into two groups: those with clinically and histopathologically proven OMDs (n = 38) and those without mucosa pathology (n = 48). The chart reviews and standardized follow-up exams (six months) were used to gather data on graft integration, wound healing, infection rates, immunosuppressive therapy, glycemic control, and smoking status. All statistical analyses were performed using SPSS Statistics version 26.0 (IBM Corp. Released 2019. IBM SPSS Statistics for Windows, Version 26.0. Armonk, NY: IBM Corp.), and a significance level of p = 0.05 was applied.

Results: OMD patients showed significantly reduced graft success rates, 27 (71.1%) vs. 44 (91.6%), p = 0.018, and an elevated incidence of wound healing complications, 11 (28.9%) vs. 5 (10.4%), p = 0.031, compared to the control group. Strong associations were found between immunosuppressive therapy (OR: 4.87, CI: 1.39-17.1, p = 0.013) and poor glycemic control (OR: 3.24, CI: 1.11-9.52, p = 0.031), as well as graft failure in the OMD group, with smoking showing a non-significant trend.

Conclusions: The prevalence of underlying OMDs was found to be associated with reduced success of bone grafts; after adjustment, failure was primarily linked to the use of immunosuppressive therapy and poor glycemic control. Successful perioperative screening, optimized metabolic management, and immunosuppressive management contribute to better outcomes.

## Introduction

Oral mucosal diseases (OMDs), including oral lichen planus, mucous membrane pemphigoid, and pemphigus vulgaris, are immune-mediated disorders that affect epithelial integrity and wound healing capacity [1]. It has been estimated that oral lichen planus alone accounts for approximately 1% of the adult population globally and is most widespread among women aged between 40 and 60 years [2]. Bone grafting is a crucial procedure in maxillofacial surgery used to rebuild alveolar defects and place dental implants [3]. Local vascularization, stable integration, and balanced immune reactions are prerequisites for successful grafting [4].

Nevertheless, OMD patients frequently need long-term immunosuppressive treatment, leading to a slowdown of wound healing and the risk of wound infections [5]. Research evidence suggests that weakened soft tissue conditions adversely influence the quality and predictive profile of bone graft incorporation [6]. Moreover, systemic variables, including imperfect glycemic control and smoking, have been demonstrated to increase healing deficits in patients with mucosal autoimmune diseases [7].

However, only a few clinical data directly compare the outcomes of bone grafting between patients with or without an underlying OMD, despite increasing insight into these interactions [8]. Therefore, there is a need to understand these associations and improve risk assessment and patient care during the oral rehabilitation process in high-risk populations. This study hypothesized that patients with OMDs had lower graft integration and more wound-healing complications at six months and that immunosuppressive therapy and poor blood sugar control were associated with graft failure. These findings may facilitate immune modulation and wound management practices to minimize complications after maxillofacial bone grafting.

The primary objective of this study was to compare six-month graph integration between patients with immune-mediated OMDs and those without OMDs undergoing maxillofacial bone grafting. Secondary objectives: (a) to compare the six-month wound healing complications and surgical site infections (SSIs); (b) to identify independent predictors of graft failure (immunosuppressive therapy, glycemic control, smoking).

## Materials and methods

This prospective cohort study was conducted from June 2023 to December 2023 to compare the clinical success of graft integration in patients with and without OMDs, with all participants followed for six months. This study was carried out in the Department of Oral and Maxillofacial Surgery of Shaikh Zayed Medical Complex, a tertiary care university hospital in Lahore (approval number: PGII/331/Sep/23).

All participants provided informed consent before enrollment. The consecutive sampling technique was used to recruit study participants who underwent autogenous or alloplastic bone grafting to enhance maxillofacial reconstruction during this period. The sample size was calculated using OpenEpi version 3.0.0 (released 2013, Atlanta, GA, USA) based on a two-sided test of two proportions: group size = 43; total n = 86; alpha = 0.05; power = 0.80; p1 = 0.90 in controls; p2 = 0.65 in OMDs; ratio = 1.0, an expected difference in graft success rates of 25% between grafts, with a significance level of 0.05, and a power of 80%. This calculation determined a minimum sample size of 86 patients (38 with OMD, 48 without OMD) [9]. All variables had less than 10% missing data and were handled by the complete-case analysis without imputation.

The inclusion criteria involved adults between 18 and 70 years of age who had received autogenous or alloplastic bone grafts for maxillofacial reconstruction, including both edentulous patients and patients undergoing grafting in association with implants, with defect sizes ranging between localized alveolar defects and a more extensive segmental type, considered appropriate to grafting, and willingness and ability to attend a minimum postoperative follow-up of six months. Patients with uncontrolled systemic conditions not related to OMDs (renal failure, cardiac disease), unreliable medical records, or limited compliance with postoperative care were excluded from the study. In the OMD group, patients needed to have a clinically and histopathologically confirmed disease (oral lichen planus, mucous membrane pemphigoid, or pemphigus vulgaris). Disease activity indices were noted in clinical charts. The controls were explicitly confirmed to be free of OMD, based on their history and intraoral examination. All eligible patients meeting the inclusion criteria were consecutively enrolled.

Patients were divided into two groups: the OMD group (participants with clinically and histopathologically proven OMDs) and the control group (patients without clinical or historical oral mucosal pathology). Bone grafts were either autogenous (from intraoral sites, such as the mandibular ramus or symphysis, or extraoral sites, like the iliac crest) or alloplastic (synthetic alternatives, such as hydroxyapatite or β-tricalcium phosphate). Three consultant oral and maxillofacial surgeons performed bone grafting procedures according to standard protocols. No blinded assessment was performed.

**Figure 1:**
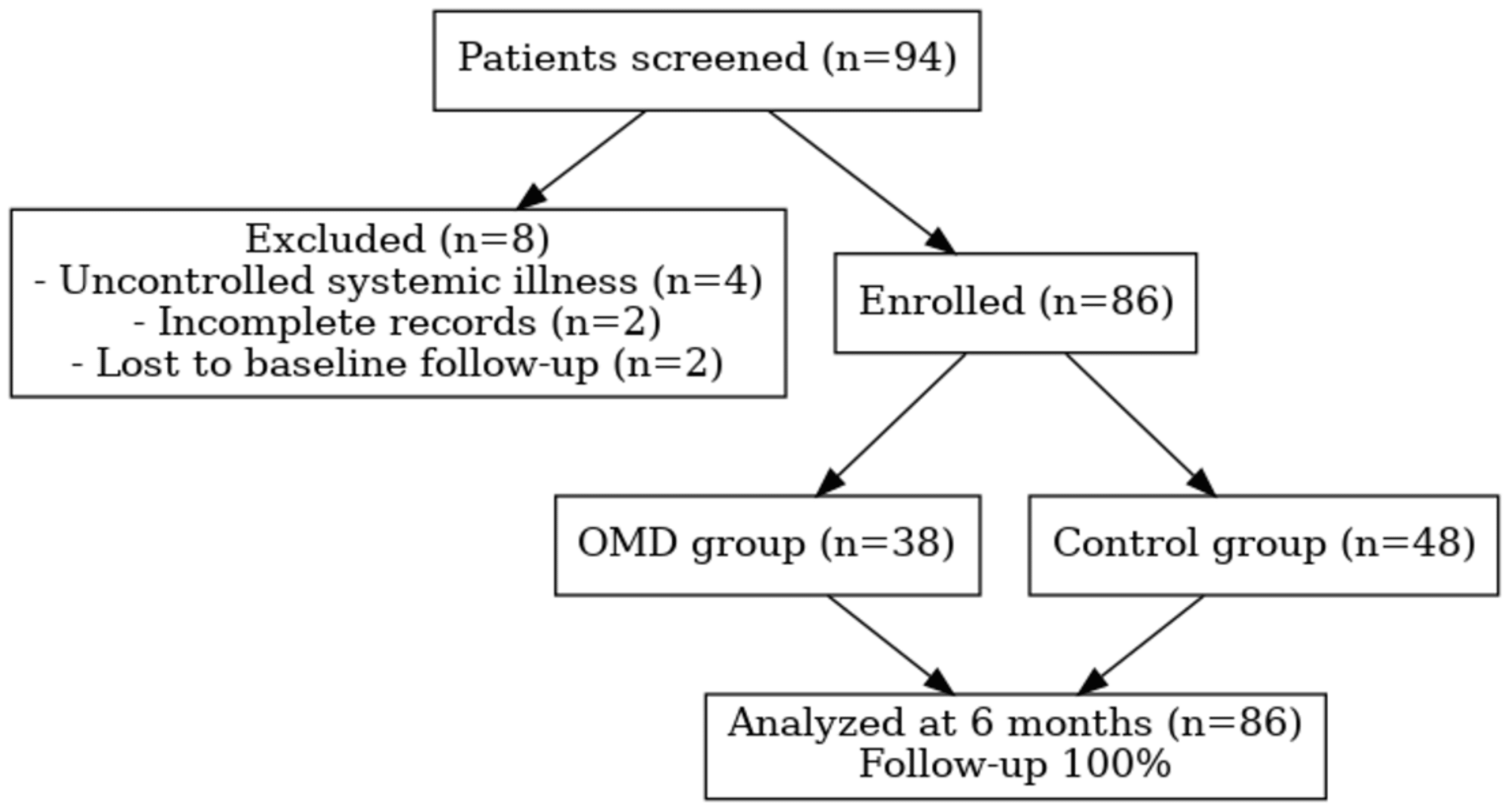
Flow diagram of patient screening n: number of participants, OMD: oral mucosal disease

A total of 94 patients were screened, but eight were excluded (four had uncontrolled systemic illness, two had incomplete records, and two were lost to baseline follow-up). The remaining 86 patients were taken for further steps, where 38 were assigned to the OMD group and 48 were assigned to the control group. All enrolled patients completed the six months of follow-up and achieved a 100% follow-up rate. Figure 1 shows the flow diagram from participant screening to follow-up.

Defects were categorized into localized alveolar (<2 cm) and segmental/maxillary (≥2 cm). Titanium mini-screw fixation was performed; collagen membranes were added for large defects, and platelet-rich fibrin was used selectively. All patients received perioperative antibiotics (amoxicillin-clavulanate 1 g twice daily or clindamycin 300 mg three times daily for five to seven days in allergic patients), NSAID analgesics, and 0.12% chlorhexidine oral rinses twice daily for 14 days. Patients with OMD on immunosuppressive therapy had prednisone-equivalent corticosteroids (10-40 mg/day), azathioprine (50-150 mg/day), or mycophenolate mofetil (1-2 g/day), with treatment durations from three months to several years. The primary outcome was early graft success, and secondary outcomes were wound healing complications and postoperative infection. Information on immunosuppressive therapy, glycemic control (as measured by HbA1c and fasting glucose), and smoking status was obtained from medical records and baseline patient interviews. HbA1c was measured within three months; an HbA1c level of ≥7% indicated poor control. Diabetes was diagnosed according to the Diagnostic Standards for Diabetes criteria. Smoking status was classified as current or former, with pack-years recorded. Immunosuppressive therapy was characterized as chronic use (≥3 months); steroid bursts within four weeks postoperatively were recorded separately. A successful graft was clinical integration without mobility or exposure, with satisfactory radiographic evidence of bone continuity at six months. Wound complications were dehiscence (>5 mm), necrosis of the mucosa, and/or persistent bleeding after 72 hours. SSI was confirmed based on the presence of purulent discharge, erythema, or swelling that necessitated antibiotic escalation and microbiological confirmation, where available.

**Figure 2:**
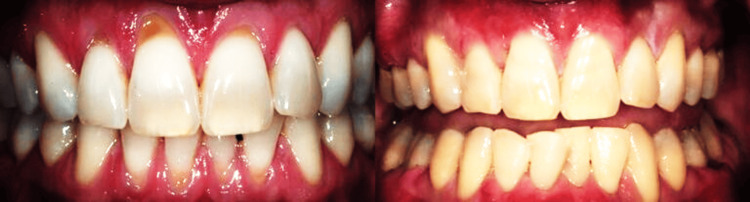
Preoperative (left) and postoperative (right) views of autogenous connective tissue grafting in the upper anterior region in control group

Preoperative (left) and postoperative (right) views of autogenous connective tissue grafting in the upper anterior region in the control group showed that the improvement can be seen in the gingival contour. Root coverage is also visible, with the healthy mucosal healing in a patient without a histopathologically proven OMD, as shown in Figure 2.

**Figure 3:**
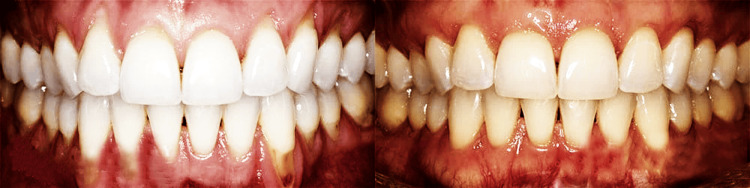
Preoperative (left) and postoperative (right) results of an autogenous pedicle graft in the lower anterior teeth in control

Preoperative (left) and postoperative (right) results of an autogenous pedicle graft in the lower anterior teeth in the control group showed an increase in the gingival thickness, improved papillary fill, and reduced root sensitivity. This patient had no histopathologically proven OMD, as shown in Figure 3.

**Figure 4:**
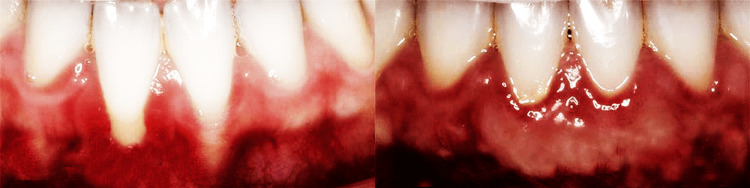
Pre- and postoperative views of alloplastic soft tissue grafting in an oral mucosal disorder group patient

Pre- and postoperative views of alloplastic soft tissue grafting in a proven oral mucosal disorder group (histopathologically confirmed case) showed that, despite baseline fragility in the mucosal lining, the graft demonstrated increased gingival volume and resilience, as shown in Figure 4.

Pre- and postoperative views of alloplastic soft tissue grafting in a proven oral mucosal disorder group (histopathologically confirmed case) showed that, despite baseline fragility in the mucosal lining, the graft demonstrated increased gingival volume and resilience, thus indicating successful integration in a histopathologically proven OMD case.

All statistical analyses were performed using SPSS Statistics version 26.0 (IBM Corp. Released 2019. IBM SPSS Statistics for Windows, Version 26.0. Armonk, NY: IBM Corp.). Besides univariable tests, multivariable logistic regression was pre-specified to obtain adjusted odds ratios (aORs) with 95% confidence intervals (CIs) of graft failure. Continuous data were presented as mean ± SD and compared using independent t-tests. The chi-square test was used to compare categorical variables, and Fisher’s exact test was applied where expected cell counts were <5. A two-tailed p-value < 0.05 was considered statistically significant.

## Results

In this study, 86 patients (38 with OMD and 48 without) were followed for six months to evaluate the primary outcome of graft integration and the secondary outcome of wound healing complications, infection, and predictors of graft failure. The graft integration rate was significantly higher in the controls (p = 0.018) compared to the patients with OMDs. Baseline and demographic characteristics of study participants are listed in Table 1. The normality was assumed and confirmed for the continuous variable (age) using the Shapiro-Wilk test (p > 0.05), which allowed for the use of parametric tests.

**Table 1:** Baseline and demographic characteristics of study population n: number of participants, OMD: oral mucosal disease, SD: standard deviation, %: percentage, *: significance at <0.05, N/A: not applicable

Parameter	OMD group (n = 38)	Control group (n = 48)	Test used	Test value	Significance (p-value)
Mean age (years) mean ± SD	49.6 ± 9.2	47.8 ± 8.7	Independent t-test	t = 1.01	0.314
Male (%)	20 (52.6%)	26 (54.1%)	Chi-square test	χ² = 0.02	0.881
Diabetic (%)	14 (36.8%)	11 (22.9%)	Chi-square test	χ² = 2.15	0.142
Smokers (%)	12 (31.6%)	12 (25.0%)	Chi-square test	χ² = 0.49	0.486
On immunosuppressives (%)	17 (44.7%)	0 (0%)	Fisher’s exact test	N/A	<0.001*

There was no significant difference in the mean age of the groups (49.6 ± 9.2 vs. 47.8 ± 8.7 years, p = 0.314). There were no significant differences in gender distribution, smoking status, and the prevalence of diabetes; however, the percentage of immunosuppressive therapy was significantly higher in the OMD group (17 (44.7%) vs. 0 (0%), p < 0.001), implying that the management of immunosuppressive treatment is an essential consideration in planning graft surgery. Table 2 summarizes the bone graft outcomes after six months.

**Table 2:** Graft outcomes after six months n: number of participants, OMD: oral mucosal disease, %: percentage, *: significance at <0.05

Outcome	OMD group (n = 38) n (%)	Control group (n = 48) n (%)	Test used	Value (χ²)	Significance (p-value)
Successful graft integration (%)	27 (71.1%)	44 (91.6%)	Chi-square test	5.62	0.018*
Wound healing complications (%)	11 (28.9%)	5 (10.4%)	Chi-square test	4.64	0.031*
Infection at graft site (%)	6 (15.8%)	3 (6.2%)	Chi-square test	2.16	0.141

The percentage of successful graft integration was significantly smaller in the OMD group, 27 (71.1%), than in controls, 44 (91.6%), p = 0.018. A higher rate of wound healing complications was encountered in patients with OMDs, 11 (28.9%) vs. 5 (10.4%), p = 0.031. OMD in the group exhibited a higher level of infections, 6 (15.8%) vs. 3 (6.2%), which did not reach statistical significance (p = 0.141), highlighting the presence of underlying mucosal disease factors that influence the healing and graft integration. To identify the independent predictors of graft failure, a pre-specified multivariable logistic regression model was developed, adjusting for all key variables. Table 3 demonstrates the multivariate regression results for independent predictors of graft failure.

**Table 3:** Multivariate regression analysis A logistic regression model was applied. *: significance at p<0.05, OMD: oral mucosal disorder, aOR: adjusted odds ratio, CI: confidence interval, HbA1c: hemoglobin A1c, %: percentage

Variable	aOR	95% CI	p-value
Immunosuppressive therapy	4.55	1.28-16.18	0.019*
Poor glycemic control (HbA1c ≥7%)	3.18	1.08-9.39	0.036*
Smoking (current/former)	1.87	0.54-6.49	0.326
OMD status	1.42	0.40-5.02	0.589

The above analysis indicated that while immunosuppressive therapy (aOR: 4.55, CI: 1.28-16.18, p = 0.019) and inadequate glycemic control (aOR: 3.18, CI: 1.08-9.39, p = 0.036) were depicting a stronger association with failure, OMD status itself was not the direct cause of graft failure. Smoking showed an insignificant trend. Table 4 presents the six-month follow-up outcomes and variables related to graft failure.

**Table 4:** Outcomes and predictors of graft failure at six months n: number of participants, %: percentage, *: significance at <0.05

Predictor variable	Graft failure n (%)	Test used	Value (χ²)	Significance (p-value)
On immunosuppressives	8 (61.5%)	Chi-square test	8.08	0.004*
Poor glycemic control	7 (53.8%)	Chi-square test	5.77	0.016*
Smoking	6 (46.1%)	Chi-square test	2.94	0.087

In OMDs, immunosuppression therapy (4.87, CI: 1.39-17.1, p = 0.013) and inadequate glycemic control (3.24, CI = 1.11-9.52, p = 0.031) were significantly associated with failure. Smoking displayed a non-significant trend, whereas graft type and defect site were not predictive. Percentages were determined among the exposed. Exposures are overlapping (e.g., some patients were both smokers and immunosuppressed). The total number of failures was 11. These outcomes suggest that a better focus on glycemic control and a prudent approach toward immunosuppressive treatment may help improve graft outcomes. Table 5 shows the sensitivity analysis of graft failure.

**Table 5:** Sensitivity analysis of graft failure OR: odds ratio, CI: confidence interval, OMD: oral mucosal disease, %: percentage, *: significance at <0.05

Restriction applied	Group (fail/total)	Failure %	Test used	OR (95% CI)	p-value
Exclude immunosuppressives	OMD	3/21 (14.3%)	Fisher’s exact	1.83 (0.37–9.03)	0.667
	Control	4/48 (8.3%)			
Exclude poor glycemic control	OMD	4/24 (16.7%)	Fisher’s exact	2.20 (0.50–9.70)	0.427
	Control	4/48 (8.3%)			
Exclude smokers	OMD	5/26 (19.2%)	Fisher’s exact	2.62 (0.64–10.77)	0.263
	Control	4/48 (8.3%)			

Excluding key risk factors, graft failure in OMD patients was observed in 3 (14.3%) without immunosuppressives, 4 (16.7%) without poor glycemic control, and 5 (19.2%) among non-smokers, compared to 4 (8.3%) among controls. None of these differences were statistically significant, supporting that the elevated crude failure in OMDs was primarily explained by systemic risk factors rather than OMD status.

## Discussion

The purpose of this study was to compare bone grafting success in patients with OMDs and in non-OMD patients over six months of follow-up in maxillofacial surgery. It also examined how systemic factors, such as immunosuppressive therapy and glycemic control, affect graft consolidation and wound healing. The findings indicated that OMDs were associated with a lower graft success rate and generated further postoperative wound complications, highlighting the role of local immune-mediated disease in regenerative outcomes.

Multivariate analysis showed that OMD was not an independent predictor of failure, indicating that systemic risk factors, including immunosuppression and poor glycemic control, are the primary independent contributors to graft outcomes. The results of the demographic and clinical data showed that confirmed OMDs, such as oral lichen planus and pemphigoid, have a significantly lower graft integration rate than controls. This is consistent with recent data supporting that mucosal autoimmune disease interferes with epithelial homeostasis and local immune balance, influencing normal wound healing and tissue regeneration [10]. A more recent study also revealed that long-term inflammation in OMDs disrupts cytokine profiles and angiogenesis, which prevents graft integration [11]. The positive relationship between immunosuppressive therapy and graft failure identified in the study is supported by a study that found the long-term use of corticosteroids or immunomodulators disrupts osteoblast differentiation and decreases bone graft stability [12]. Similarly, the results indicated that the risk of graft failure was significantly higher when glycemic control was poor, which confirms findings by previous literature that hyperglycemia restricts angiogenesis and reduces new bone formation [13].

Interestingly, smoking did not represent a statistically significant factor in our cohort, which is contrary to the results of a study that found smoking to be an independent predictor of peri-implant bone loss [14]. This difference may be caused by an insufficient sample size of smokers or variations in the frequency of smoking that this study did not stratify. Recent developments, including platelet-rich fibrin and bioactive scaffolds, are promising for graft healing improvement, especially in patients with compromised mucosal health [15,16]. These outcomes suggest that preoperative screening, stabilization of the disease, and interdisciplinary collaboration with oral medicine doctors can significantly improve patient outcomes regarding OMDs [17]. Possible measures to reduce complications and increase surgical success include adjustment of immunosuppressive regimens, good metabolic control, and the use of biomimetic grafting material [18,19].

The limitations of the present study include a single-center design that may potentially impact generalizability and create bias in selection. Factors, including OMD activity indices, extent of disease, specific classification of graft defects, and surgeon variability, were not measured, which may have affected outcomes and remain as sources of confounding. Defect size/site, graft material, use of membrane, surgeon identity, and activity/severity of OMD were recorded but not stratified in outcome analyses; other granular details (e.g., specific diseases, disease extent indices) were not quantified. Moreover, the six-month follow-up provides early outcomes, but no long-term graft survival. These factors moderate the solidity of inference, and the results should be taken with caution. The limitations imply that the evidence should be considered observational and hypothesis-generating. However, its results emphasize the importance of risk assessment and perioperative optimization, such as disease stabilization, vigilant immunosuppressive therapy, and tight glycemic control, to mitigate the risk of graft failure.

## Conclusions

The presence of OMD alone was not an independent predictor once graft type and defect site were considered within six months in OMD patients; however, these associations are affected by immunosuppressive treatment and metabolic status. The study highlights the importance of rigorous preoperative assessment and optimization with emphasis on disease stabilization, careful immunosuppressive management, and strict metabolic control. Given the observational design, causality cannot be inferred, and results should be regarded as hypothesis-generating.

The implementation of individual approaches to treatment, multidisciplinary care, and the investigation of innovative methods of regeneration can contribute to the increased effectiveness of surgical interventions and long-term recovery among these high-risk patients.
